# Hoxa5 increases mitochondrial apoptosis by inhibiting Akt/mTORC1/S6K1 pathway in mice white adipocytes

**DOI:** 10.18632/oncotarget.20521

**Published:** 2017-08-24

**Authors:** Fei Feng, Qian Ren, Song Wu, Muhammad Saeed, Chao Sun

**Affiliations:** ^1^ College of Animal Science and Technology, Northwest A&F University, Yangling, Shaanxi 712100, China

**Keywords:** Hoxa5, white adipocytes, mitochondrial apoptosis, mice, Akt/mTORC1 pathway

## Abstract

Homeobox A5(*Hoxa5*), a member of the *Hox* family, plays a important role in the regulation of proliferation and apoptosis in cancer cells. The dysregulation of the adipocyte apoptosis *in vivo* leads to obesity and metabolic disorders. However, the effects of Hoxa5 on adipocyte apoptosis are still unknown. In this study, palmitic acid (PA) significantly increased the mRNA level of *Hoxa5* and triggered white adipocyte apoptosis *in vivo* and *in vitro*. Further analysis revealed that Hoxa5 enhanced the early and late apoptotic cells and fragmentation of genomic DNA in adipocytes from inguinal white adipose tissue (iWAT) of mice. Moreover, Hoxa5 aggravated white adipocyte apoptosis through mitochondrial pathway rather than endoplasmic reticulum stress (ERS)-induced or death receptor (DR)-mediated pathway. Our data also confirmed that Hoxa5 promoted mitochondrial apoptosis pathway by elevating the transcription activity of *Bax* and inhibiting the protein kinase B (Akt)/mammalian target of rapamycin complex 1 (mTORC1) signaling pathway. In summary, these findings revealed a novel mechanism that linked Hoxa5 to white adipocyte apoptosis, which provided some potential possibilities to prevent and treat obesity and some metabolic diseases.

## INTRODUCTION

Hoxa5 protein is a member of the Hox protein family and is an important transcription factor which plays a definitive role in patterning the cells and tissues, especially the anteroposterior body axis during embryogenesis [1–3]. And Hoxa5 is known to be associated with the process of cell proliferation, migration and apoptosis in cancer cells [4–6]. Previous studies have shown that abnormal expression of some *Hox* genes in multiple cancer types [7]. Raman et al. (2000) have found that Hoxa5 expression is low in breast cancer cell lines and forced-expression of Hoxa5 induces apoptosis [8, 9]. Moreover, Hoxa5 could increase apoptosis by p53-dependent or p53-independent pathway, especially in cancer cells, such as breast cancer cells, epithelial cancer cells, lipoma cells and lung cancer cells [10, 11]. In addition, retinoid acid (RA) bound to retinoid acid receptors (RARs) and transcriptionally activated *Hoxa5*, which was important for RA-mediated apoptosis increase and proliferation inhibition in a variety of breast cancer cells [12, 13].

Akt/mTOR signaling pathway is essential for multiple cellular processes, such as cell proliferation, apoptosis, cell migration, differentiation and glucose metabolism [14–17]. Current evidences suggest that Akt signaling pathway plays a critical role in regulating *Hox* gene expression during RA-induced apoptosis [18, 19]. Knockdown of some *Hox* genes inhibited activation of extracellular regulated protein kinases 1/2 (ERK1/2) and Akt pathway in prostate cancer cells [20]. These findings implied that Hoxa5 might act as an important mediator of several pathways in coordinating apoptosis.

Obesity is a complex chronic disease and it increases risk for human health by causing type 2 diabetes, fatty liver, hyperlipidemia and other metabolic disorders. Moreover, obesity is determined by the number and volume of adipocytes in adipose tissue, which depends on the balance between cell survival and apoptosis [21–23]. Previous studies have found that PA induced dysfunction and apoptosis by activating reactive oxygen species (ROS) generation in pancreatic β-cells and podocytes. And PA could trigger mouse preadipocyte apoptosis by increasing cysteinyl aspartate specific proteinase (caspase) cascade reaction [24–26]. Some researchers have found that in epididymal adipose tissue of mice fed with a high-fat diet (HFD), *Hoxa5* undergoes dynamic DNA methylation and transcriptional repression, which may represent a potential way to quantify obesity response to nutritional intervention [27]. However, recent studies about Hoxa5 mainly focus on cancer cells and the precise molecular mechanisms involved in the regulation of Hoxa5 on adipocyte apoptosis are unclear.

In this study, we investigated the effects of Hoxa5 on white adipocyte apoptosis. We have shown that Hoxa5 increased apoptosis by inhibiting Akt/mTORC1 signaling pathway in white adipocytes of mice. In addition, *Bax*, as a target of Hoxa5, was very important in Hoxa5-induced apoptosis through mitochondrial pathway. And these findings might open new therapeutic possibilities against obesity and type 2 diabetes.

## RESULTS

### PA triggered apoptosis along with the increased *Hoxa5* mRNA level in white adipose tissue of mice

**Figure 1:**
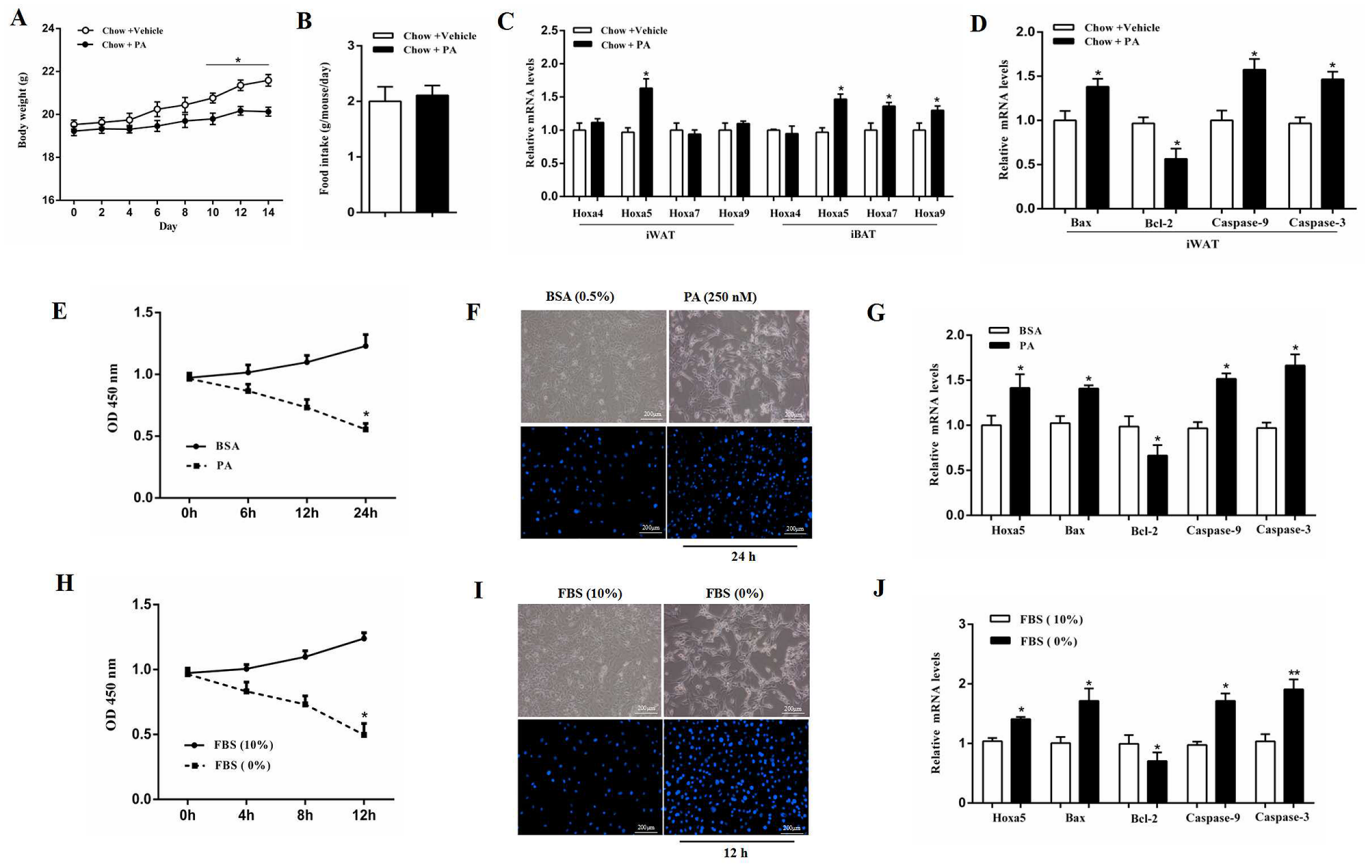
PA triggered apoptosis along with the increased *Hoxa5* mRNA level in white adipose tissue of mice **(A)** Body weight of mice fed on chow diet after palmitate acid (PA, 1 μg/g) injection per 2 days during 2 weeks (n = 6). **(B)** Food intake of mice fed on chow diet after PA injection (n = 6). **(C, D)** Relative mRNA levels of *Hoxa4*, *Hoxa5*, *Hoxa7* and *Hoxa9* in iBAT and iWAT (C) andrelative mRNA levels of *Bax*, *Bcl-2*, *Caspase-9* and *Caspase-3* in iWAT (D) of mice fed on chow diet after PA injection on the 14^th^ day (n = 6). **(E)** Relative OD values for cell viability of white adipocytes after 250 nM PA incubation for 6, 12 and 24 h (n = 6). **(F)** Hoechst 33258 staining of white adipocyte apoptosis after PA incubation for 24 h. Scale bar: 200 μm (n = 3). **(G)** Relative mRNA levels of *Hoxa5*, *Bax*, *Bcl-2*, *Caspase-9* and *Caspase-3* in white adipocytes after PA incubation for 24 h (n = 6). **(H)** Relative OD values for cell viability of white adipocytes after serum starvation for 4, 8 and 12 h (n = 6). **(I)** Hoechst 33258 staining of white adipocyte apoptosis after serum starvation for 12 h. Scale bar: 200 μm (n = 3). **(J)** Relative mRNA levels of *Hoxa5*, *Bax*, *Bcl-2*, *Caspase-9* and *Caspase-3* in white adipocytes after serum starvation for 12 h (n = 6). Values are mean ± SEM. ^*^P < 0.05, ^**^P < 0.01 compared with the control.

Our result showed that body weight was decreased after intraperitoneal injection of PA (*P* < 0.05), while there was no difference in food intake (Figure 1A and 1B). PA treatment increased the mRNA level of *Hoxa5* in both inguinal white adipose tissue (iWAT) and interscapular brown adipose tissue (iBAT) (*P* < 0.05), but did not change the expression of *Hoxa4*, *Hoxa7* and *Hoxa9* in iWAT compared with those in iBAT (Figure 1C). We then measured the mRNA levels of *Bax*, *Bcl-2*, *Caspase-9* and *Caspase-3*, and data showed PA significantly increased the mRNA levels of pro-apoptotic genes (*P* < 0.05) (Figure 1D). Additionally, mice white adipocytes from iWAT were incubated with 250 nM PA for 24 h. Our results demonstrated PA reduced adipocyte viability (*P* < 0.05) and increased the number of apoptotic cells (Figure 1E and 1F). Interestingly, PA also elevated the mRNA level of *Hoxa5* (*P* < 0.05) and triggered white adipocyte apoptosis by promoting the expression of *Bax*, *Caspase-9* and *Caspase-3*, but suppressing *Bcl-2* (*P* < 0.05) (Figure 1G). Similarly, serum-free medium treatment also induced white adipocyte apoptosis and increased the mRNA level of *Hoxa5* (*P* < 0.05) (Figure 1H-1J). Thus, PA triggered apoptosis in white adipose tissue along with a higher level of *Hoxa5*. So, we speculated Hoxa5 might increase white adipocytes apoptosis in mice.

### Hoxa5 increased apoptosis of white adipocytes

**Figure 2:**
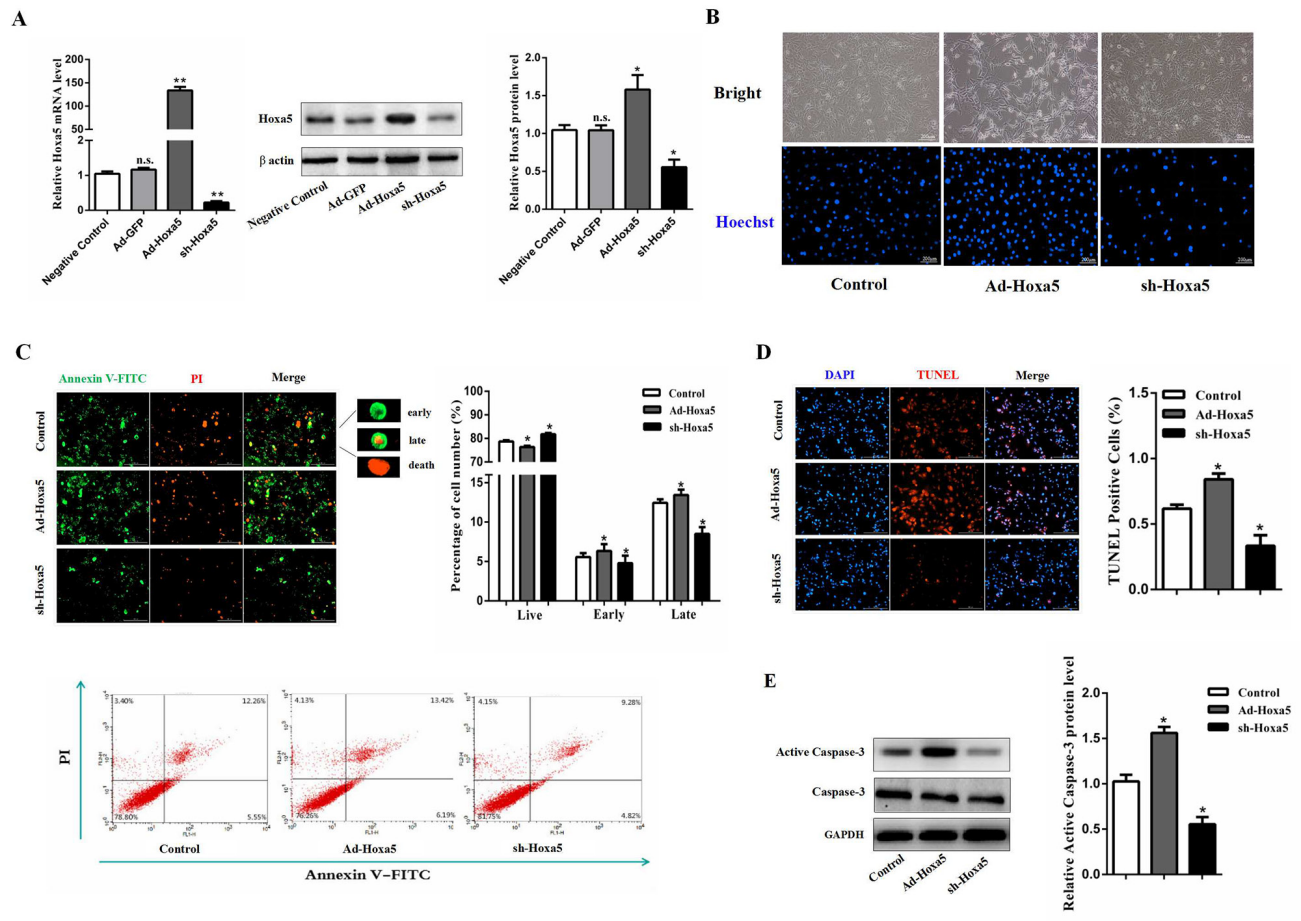
Hoxa5 increased apoptosis of white adipocytes White adipocytes were pretreated with either Ad-Hoxa5 or sh-Hoxa5. **(A)** Efficiencies of Ad-Hoxa5 or sh-Hoxa5 in white adipocytes (n = 6). **(B)** Hoechst 33258 staining of white adipocyte apoptosis. Scale bar: 200 μm (n = 3). **(C)** Annexin V-FITC/PI double staining and flow cytometry analysis of white adipocyte apoptosis. Scale bar: 200 μm (n = 3). **(D)** TUNEL staining of DNA fragments in white adipocytes. Scale bar: 200 μm (n = 3). **(E)** Relative protein level of active Caspase-3 in white adipocytes (n = 3). Values are mean ± SEM. ^*^P < 0.05, ^**^P < 0.01 compared with the control.

Mice white adipocytes were infected with adenovirus overexpression vector of Hoxa5 (Ad-Hoxa5) or adenovirus interference vector of Hoxa5 (sh-Hoxa5). We found there were no difference between Ad-GFP and negative control in Hoxa5 mRNA and protein level (*P* > 0.05). Compared with the control group, Ad-Hoxa5 and sh-Hoxa5 were functional (Figure 2A). As shown in Figure 2B, Ad-Hoxa5 elevated the number of apoptotic adipocytes with Hoechst staining. In addition, Annexin V/PI staining and an analysis by flow cytometry showed a higher percentage of apoptotic cells in the early and late stage after Ad-Hoxa5 treatment as compared with control group (*P* < 0.05) (Figure 2C). Further, we detected the fragments of genome DNA in nucleus with TUNEL staining, indicating that overexpression of Hoxa5 increased the number of TUNEL-positive cells (*P* < 0.05), while the knockdown group had less positive cells (*P* < 0.05) (Figure 2D). Western blot analysis showed that Hoxa5 also enhanced the protein level of active Caspase-3 (*P* < 0.05) (Figure 2E). Together, these data suggested that Hoxa5 suppressed survival and promoted the apoptosis process of white adipocytes.

### Hoxa5 promoted apoptosis through mitochondrial pathway in white adipocytes

**Figure 3:**
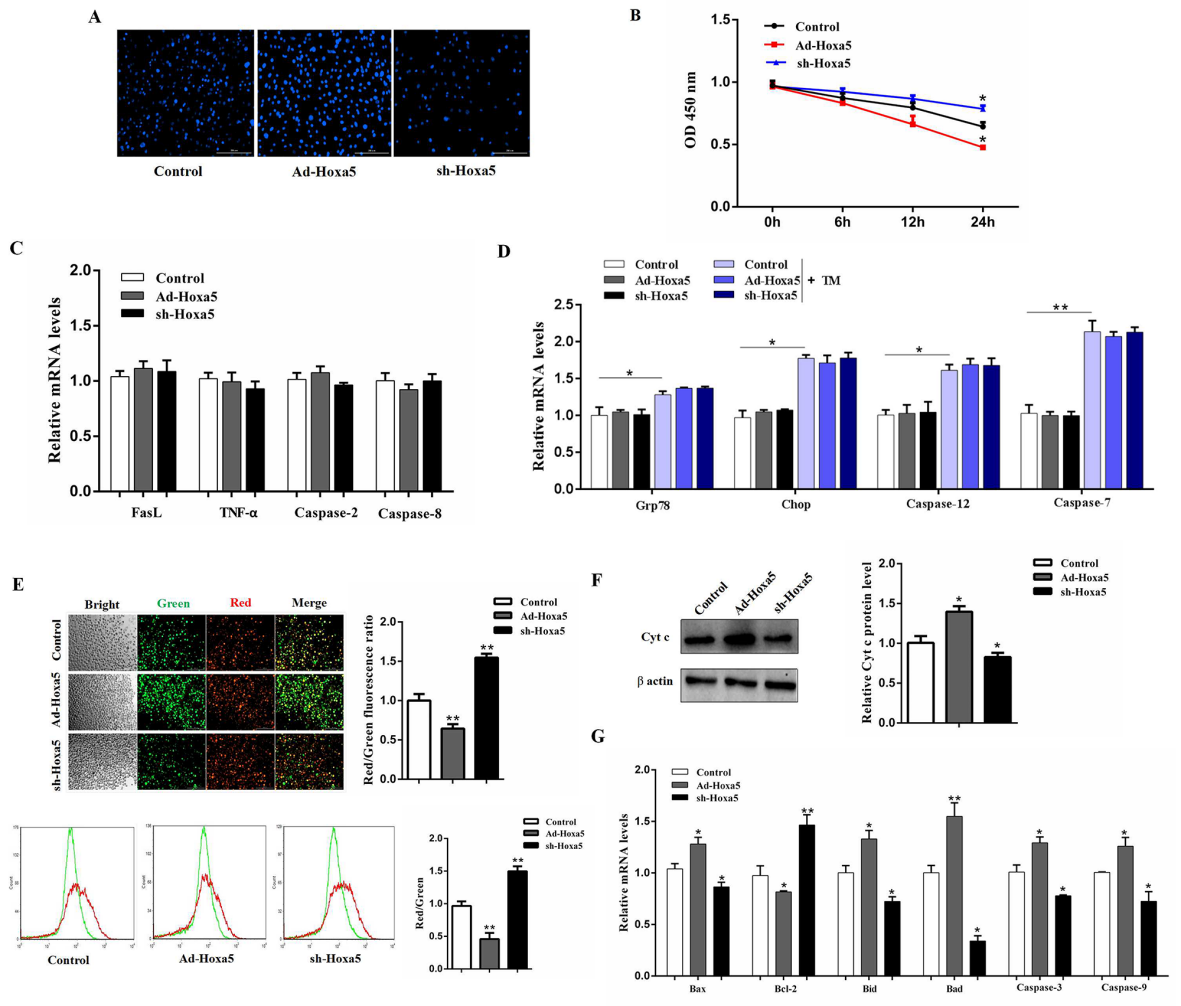
Hoxa5 promoted apoptosis through mitochondrial pathway in white adipocytes White adipocytes were pretreated with either Ad-Hoxa5 or sh-Hoxa5. **(A)** Hoechst 33258 staining of white adipocyte apoptosis for 24 h. Scale bar: 200 μm (n = 3). **(B)** Relative OD values for cell viability of white adipocytes for 6, 12 and 24 h (n = 6). **(C, D)** Relative mRNA levels of *FasL*, *TNF-α*, *Caspase-2*, *Caspase-8* **(C)**, *Grp78*, *Chop*, *Caspase-12* and *Caspase-7* **(D)** (n = 6). **(E)** JC-1 staining and flow cytometry analysis for mitochondrial membrane potential of white adipocytes. Scale bar: 200 μm (n = 3). **(F)** Relative protein level of Cytochrome-c (Cyt-c) in white adipocytes (n = 3). **(G)** Relative mRNA levels of *Bax*, *Bcl-2*, *Bid*, *Bad*, *caspase-3* and *caspase-9* in white adipocytes (n = 6). Values are mean ± SEM. ^*^P < 0.05, ^**^P < 0.01 compared with the control.

Next, we addressed how Hoxa5 increased apoptosis in mice white adipocytes. Hoechst staining analysis showed Ad-Hoxa5 treatment markedly enhanced apoptotic cells (Figure 3A). And as expected, cell viability was decreased in Ad-Hoxa5 group (*P* < 0.05) (Figure 3B). However, further analysis demonstrated there were no change in mRNA levels of *FasL*, *TNF-α*, *caspase-2* and *caspase-8* of white adipocytes (*P* > 0.05), which were key genes regulating the DR-mediated apoptosis pathway (Figure 3C). And our data showed ERS marker genes *Grp78* and *Chop* were both elevated after tunicamycin (TM) treatment (*P* < 0.05) and further measurements indicated that TM treatment triggered apoptosis of white adipocytes by increasing the mRNA levels of *Caspase-12* (*P* < 0.05) and *Caspase-7* (*P* < 0.01). Interestingly, Hoxa5 did not change these marker genes of the ERS-induced apoptotic pathway (*P* *>* 0.05) (Figure 3D). Then, to examine how Hoxa5 increased apoptosis in mice white adipocytes, we tested mitochondrial membrane potential. JC-1 staining and flow cytometry analysis showed that Ad-Hoxa5 treatment lowered the ratio of red/green fluorescence intensity compared with control group (*P* < 0.01) (Figure 3E). Moreover, Ad-Hoxa5 treatment significantly increased (*P* < 0.05) Cytochrome-c (Cyt-c) protein (Figure 3F), which bound to apoptotic protease activating facter-1 (Apaf-1), forming an apoptosome complex with procaspase-9 and subsequently activated downstream effector caspases to induce apoptosis. Those changes were correlated with the increase of mitochondrial pro-apoptotic markers *Bax* (*P* < 0.05), *Bid* (*P* < 0.05), *Bad* (*P* < 0.01), *Caspase-9* (*P* < 0.05), *Caspase-3* (*P* < 0.05) and the reduction of mitochondrial anti-apoptotic marker *Bcl-2* (*P* < 0.05) after forcing expression of Hoxa5 (Figure 3G). Our above data collectively revealed Hoxa5 increased adipocyte apoptosis through mitochondrial pathway, but not other pathways, such as ERS-induced and DR-mediated pathway.

### Hoxa5 aggravated PA- and serum starvation-induced white adipocyte apoptosis

**Figure 4:**
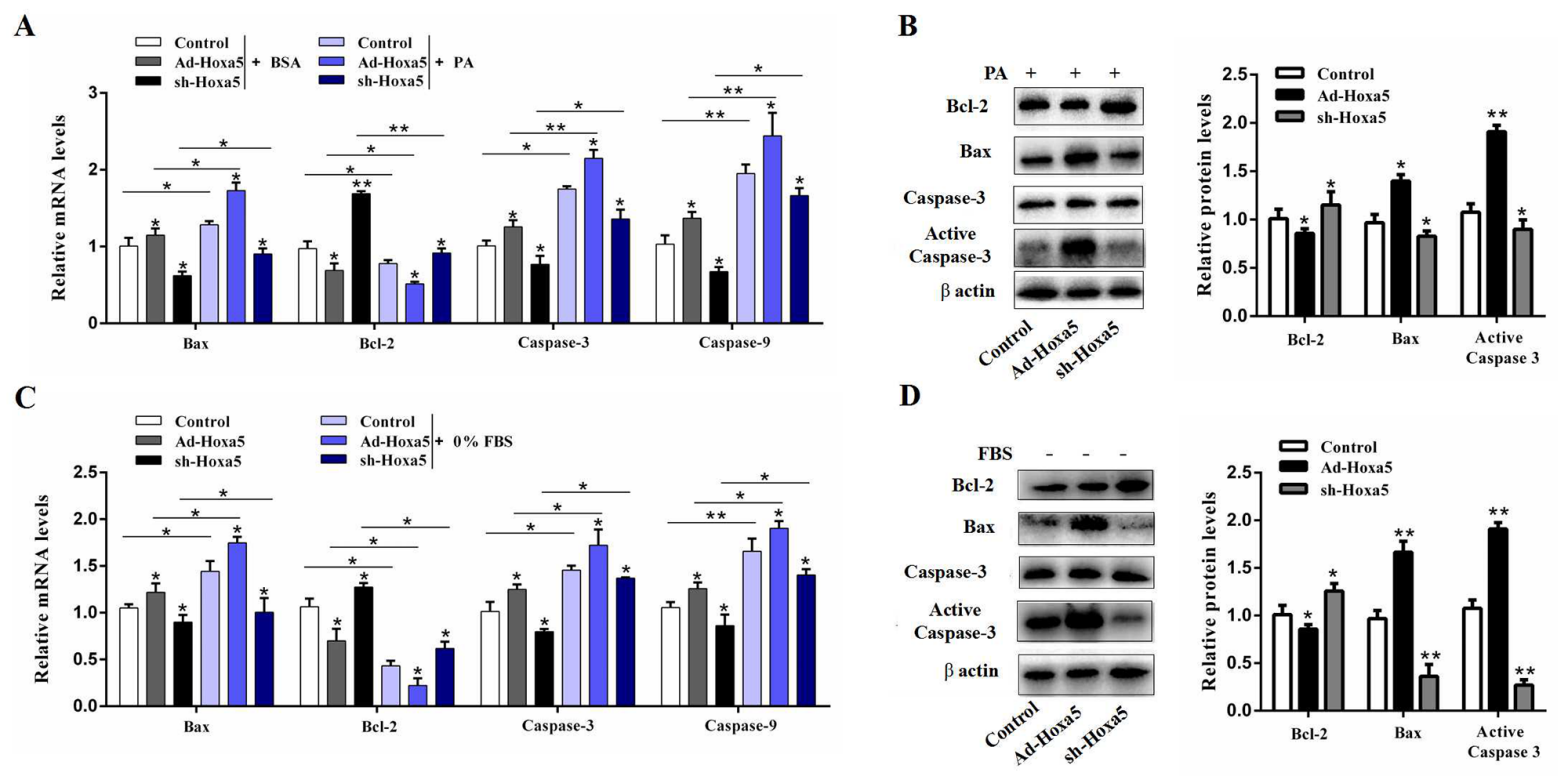
Hoxa5 aggravated PA- and serum starvation-induced white adipocyte apoptosis White adipocytes were pretreated with either Ad-Hoxa5 or sh-Hoxa5. **(A, B)** Relative mRNA levels of *Bax*, *Bcl-2*, *Caspase-3* and *Caspase-9* (n = 6) (A) and protein levels of Bax, Bcl-2, active Caspase-3(n = 3) (B) in white adipocytes with 250 nM PA treatment for 24 h. **(C, D)** Relative mRNA levels of *Bax*, *Bcl-2*, *Caspase-3* and *Caspase-9* (n = 6) (C) and protein levels of Bax, Bcl-2, active Caspase-3 (n = 3) (D) in white adipocytes with serum starvation treatment for 12 h. Values are mean ± SEM. ^*^P < 0.05, ^**^P < 0.01 compared with the control.

We then studied the function of Hoxa5 in two different models of apoptosis to further verify the pro-apoptotic effects of Hoxa5. White adipocytes were incubated with 250 nM PA for 24 h or serum-free medium for 12 h. Our data demonstrated that PA increased the mRNA levels of *Bax*, *Caspase-3* and *Caspase-9*, but reduced *Bcl-2* (*P* < 0.05 or *P* < 0.01). The combination with forced expression of Hoxa5 also increased the mRNA and protein level of pro-apoptotic genes (*P* < 0.05) (Figure 4A and 4B). Similarly, overexpression of Hoxa5 aggravated starvation-induced adipocyte apoptosis as well. A higher mRNA (*P* < 0.05) and protein level of Bax (*P* < 0.01), increased protein level of active caspase-3 (*P* < 0.01) and reduced mRNA and protein level of Bcl-2 (*P* < 0.05) indicated that adipocyte apoptosis was successfully aggravated by a high expression of Hoxa5 (Figure 4C and 4D). The above data demonstrated Hoxa5 promoted the process of PA- and serum starvation-induced apoptosis in white adipocytes, further verifying the pro-apoptotic effect of Hoxa5.

### Hoxa5 promoted apoptosis by elevating *Bax* transcription in white adipocytes

**Figure 5:**
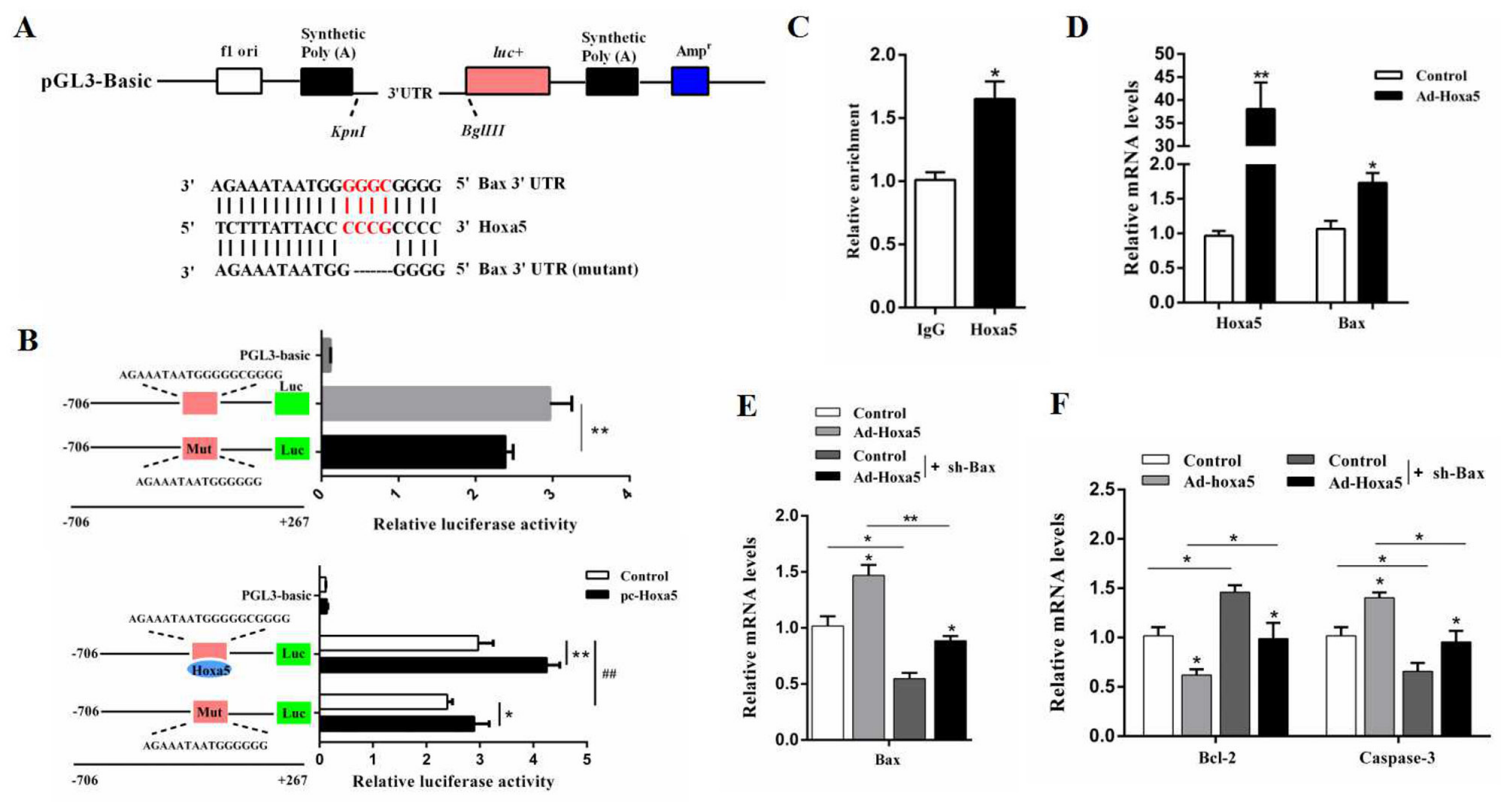
Hoxa5 promoted apoptosis by elevating *Bax* transcription in white adipocytes **(A)** The construction of the luciferase (Luc) expression vector fused to the 3’UTR and predicted target sites between Hoxa5 and mouse *Bax* 3’UTR. **(B)** Dual luciferase reporter assay of Hoxa5 and *Bax*. HEK293T cells were transfected with PGL3-basic (control), PGL3-Bax or PGL3-Bax-mutant plasmids (top); HEK293T cells were transfected with PGL3-basic (control), PGL3-Bax or PGL3-Bax-mutant plasmids together with pc-Hoxa5 (bottom) (n = 6). **(C)** ChIP analysis between Hoxa5 and *Bax* (n = 3). **(D)** Relative mRNA levels of *Hoxa5* and *Bax* with Ad-Hoxa5 infection of white adipocytes (n = 6). **(E, F)** Relative mRNA levels of and *Bax* **(E)**, *Bcl-2* and *Caspase-3* **(F)** of white adipocytes after co-transfection with Ad-Hoxa5 and sh-Bax (n = 6). Luciferase activity was corrected for Renilla luciferase activity and normalized to the control activity. PGL3-Bax: fragment of Bax promoter region fused to a luciferase reporter, PGL3-Bax-mutant: fragment of mutant *Bax* promoter fused to a luciferase reporter. Values are mean ± SEM. ^*, #^*P* < 0.05, ^**, ##^ *P* < 0.01 compared with the control.

Analysis of our online prediction demonstrated that the *Bax* promoter contained an important binding site, 129 bp - 111 bp upstream of the initiation codon of *Bax*, which was the potential target of Hoxa5 (Figure 5A). Mutation of the binding site in the *Bax* promoter reduced the *Bax* transcription activity (*P* < 0.01) (Figure 5B). We then treated cells with the overexpression of Hoxa5, and the result showed that Hoxa5 overtly enhanced the level of *Bax* transcription (*P* < 0.01) (Figure 5B). Additionally, the transcriptional regulation between Hoxa5 and *Bax* was also verified by the ChIP measurement (*P* < 0.05) (Figure 5C). And Figure 5D showed that Ad-Hoxa5 significantly increased the mRNA levels of *Hoxa5* (*P* < 0.01) and *Bax* (*P* < 0.05) in white adipocytes. Then, further investigation found that overexpression of Hoxa5 elevated the mRNA levels of *Bax* and *Caspase-3* (*P* < 0.05), but reduced *Bcl-2* (*P* < 0.05) in Bax interference group (Figure 5E and 5F). Together, the data strongly suggested Hoxa5 controlled apoptosis by elevating *Bax* transcription level in white adipocytes.

### Hoxa5 increased white adipocyte apoptosis via the Akt/mTORC1 pathway

**Figure 6:**
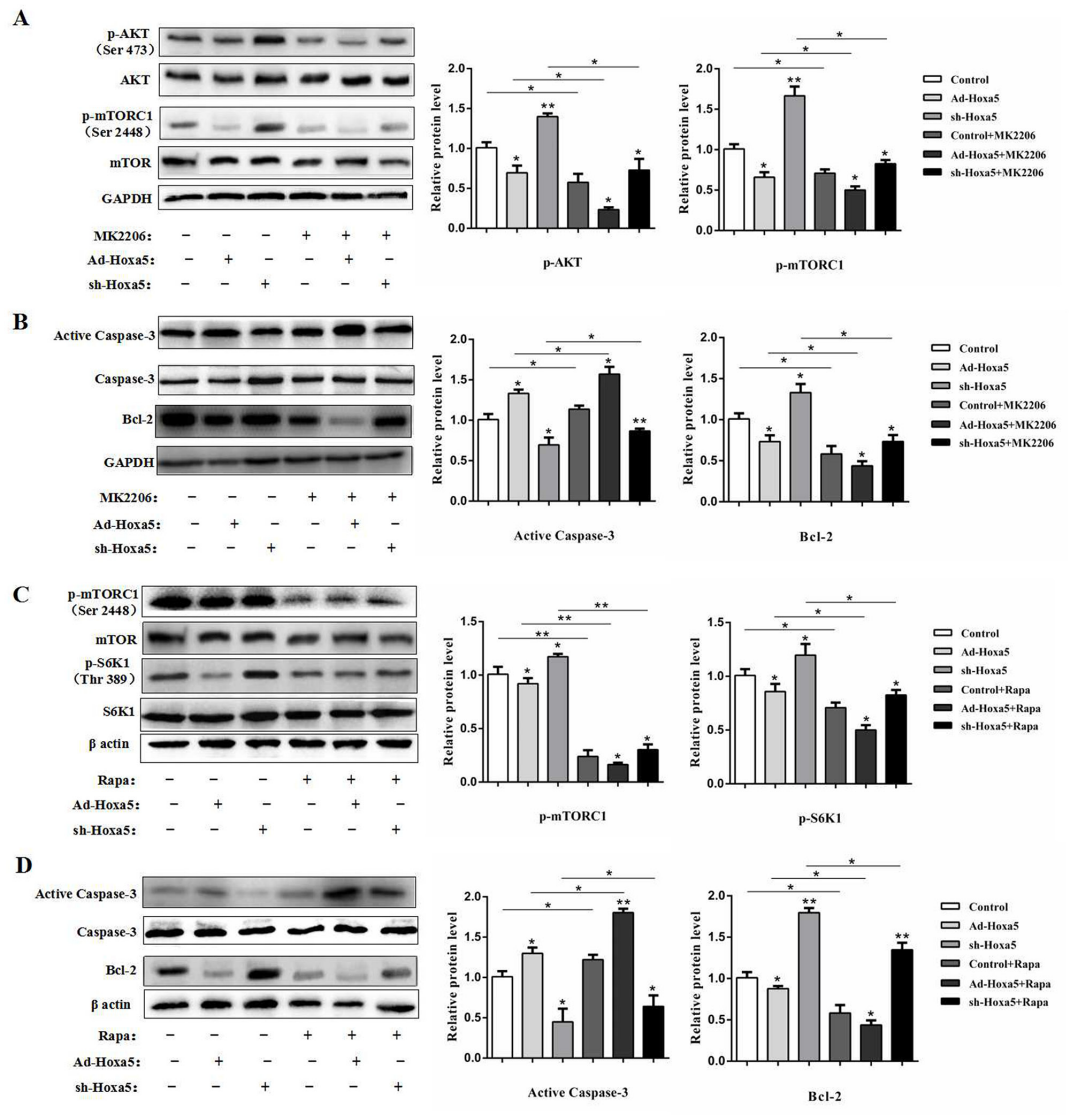
Hoxa5 increased white adipocyte apoptosis via the Akt/mTORC1 pathway White adipocytes were pretreated with Ad-Hoxa5 or sh-Hoxa5, MK2206 or rapamycin. **(A)** Relative phosphorylation levels of Akt^Ser473^ and mTORC1^Ser2448^ with or without MK2206 in each group of white adipocytes (n = 3). **(B)** Relative protein levels of active Caspase-3 and Bcl-2 with or without MK2206 in each group of white adipocytes (n = 3). **(C)** Relative phosphorylation levels of mTORC1^Ser2448^ and S6K1^Thr389^ with or without rapamycin in each group of white adipocytes (n = 3). **(D)** Relative protein levels of active Caspase-3 and Bcl-2 with or without rapamycin in each group of white adipocytes (n = 3). Values are mean ± SEM. ^*^P < 0.05, ^**^P < 0.01 compared with the control.

To further dissect the possible molecular mechanism of Hoxa5 on the adipocyte apoptosis, we determined whether Hoxa5 regulate the mTOR pathway in white adipocytes. We found reduced phosphorylation levels of Akt at Ser473, mTORC1 at Ser2448 and ribosome protein subunit 6 kinase 1 (S6K1) at Thr389 in the Hoxa5 forced expression group compared to the control (*P* < 0.05) (Figure 6A and 6C), suggesting that Hoxa5 disturbed the mTORC1 signaling pathway, involving the upstream and downstream targets. Along with the inactivation of mTORC1 signaling pathways, Hoxa5 increased the protein levels of active caspase-3 and reduced Bcl-2 (*P* < 0.05) (Figure 6B and 6D). Thus, these results suggest that Hoxa5 triggered the adipocyte apoptosis by inhibiting mTORC1 signaling pathway. We then further studied whether these effects were affected by Akt/mTORC1 signaling. Treatment with the Akt inhibitor MK2206 significantly reduced the phosphorylation level of Akt at Ser473 (*P* < 0.05) (Figure 6A). MK2206 also decreased the phosphorylation level of the downstream target, mTORC1, at Ser2448 (*P* < 0.05) and the protein level of Bcl-2 (*P* < 0.05), but elevated activity of cleaved caspase-3 (*P* < 0.05) (Figure 6A and 6B). So, Hoxa5 aggravated the effect of MK2206 on apoptosis. Moreover, we observed a lower phosphorylation levels of mTORC1 at Ser2448 (*P* < 0.01) and S6K1 at Thr389 (*P* < 0.05) when we treated adipocytes with rapamycin, a specific phosphorylation inhibitor of mTORC1 (Figure 6C). And rapamycin increased the activity of cleaved caspase-3 (*P* < 0.05) and reduced the protein level of Bcl-2 (*P* < 0.05) (Figure 6D). Similarly, Hoxa5 aggravated the effect of rapamycin on apoptosis. Collectively, these data provided evidence to suggest that Hoxa5 increased apoptosis of white adipocytes by inhibiting the Akt/mTORC1/S6K1 signaling pathway.

### Hoxa5 promoted PA-induced apoptosis in white adipose tissue of mice

**Figure 7:**
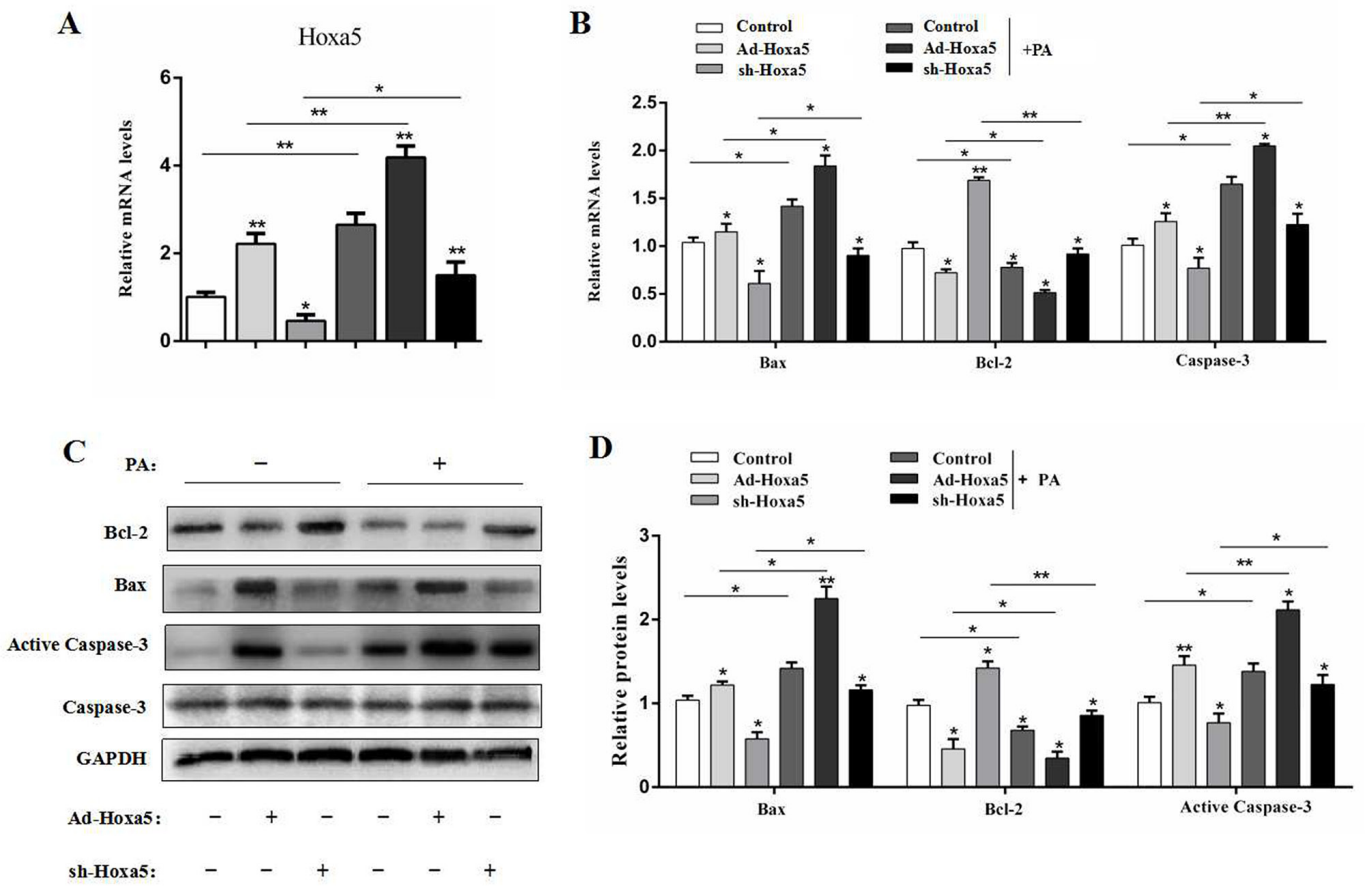
Hoxa5 promoted PA-induced apoptosis in white adipose tissue of mice Seven-week-old mice were intraperitoneally injected either PBS or PA, Ad-Hoxa5 or Sh-Hoxa5. **(A, B)** Relative mRNA level of *Hoxa5* (A), *Bax*, *Bcl-2* and *Caspase-3* (B) in the iWAT of mice after Ad-Hoxa5 or sh-Hoxa5 and PA or PBS injection once a day for 6 days (n = 6). **(C, D)** Relative protein levels of Bax, Bcl-2 and Active Caspase-3 in the iWAT of mice after Ad-Hoxa5 or sh-Hoxa5 and PA or PBS injection once a day for 6 days (n = 3). Values are mean ± SEM. ^*^P < 0.05, ^**^P < 0.01 compared with the control.

We next intraperitoneally injected Ad-Hoxa5 or sh-Hoxa5 to investigate the role of Hoxa5 in PA-induced adipocyte apoptosis in mice. Data showed PA injection markedly promoted the mRNA level of *Hoxa5* in iWAT (*P* < 0.01). And, Ad-Hoxa5 injection also elevated *Hoxa5* mRNA level effectively (*P* < 0.01) (Figure 7A). We then measured the mRNA and protein levels of Bax, Bcl-2 and Caspase-3. And as expected, PA significantly increased apoptosis in iWAT (*P* < 0.05). However, the apoptosis was alleviated after sh-Hoxa5 injection, and aggravated in Ad-Hoxa5 group (Figure 7B-7D). Thus, we concluded Hoxa5 aggravated PA-induced apoptosis in iWAT of mice.

## DISCUSSION

Excess lipid accumulation, caused by the increased number and volume of adipocytes directly, contributes to obesity, type 2 diabetes, fatty liver and other metabolic syndromes [28–30]. In addition to nutritional levels and environmental conditions, the unbalance between cell proliferation and apoptosis of adipocytes might lead to obesity [26].

Apoptosis, or programmed cell death, eliminates unneeded and dangerous cells timely and effectively throughout the lifespan of multicellular organisms [31, 32]. In recent years, many studies focused on the exogenous apoptotic pathway, that is, the DR-mediated pathway and the endogenous apoptotic pathway, that is, the mitochondrial pathway. In addition, they also found other ways involved in regulation of apoptosis, such as ERS-induced and cell cycle arrest-induced apoptosis [33, 34]. Interestingly, in this study, we found Hoxa5 triggered apoptosis through mitochondrial pathway, but not other ways. In the mammal, the Bcl-2 family proteins play a crucial role in regulating caspase activation through mitochondrial pathway. Some external environmental factors stimulate cells to response to apoptosis signals, resulting in the imbalance of pro- and anti-apoptotic Bcl-2 family members, especially affecting Bax, Bcl-2, Bad and Bid on the mitochondrial membrane [35, 36]. Then mitochondrial proteins including Cytochrome-c (Cyt-c) and second mitochondria-derived activator of caspases (Smac) are released into the cytoplasm. Cyt-c binds to Apaf-1, forming an apoptosome complex with procaspase-9 and Caspase-9 is activated. Subsequently, the activation of downstream caspases results in apoptosis. Parallelly, Smac protein binds to the inhibitor of apoptosis protein (IAP), suppressing the inhibitory effect of caspase activity, which promotes the process of apoptosis [34, 37]. Here, we found that Hoxa5 significantly increased the early and late apoptotic cells, decreased the mitochondrial membrane potential and aggravated nuclear genomic DNA fragmentation. Moreover, data showed that Hoxa5 enhanced the expression of pro-apoptotic genes involved in mitochondrial pathway, such as Bax, Bim, Bid, cleaved caspase-9 and cleaved caspase-3. Besides, results also revealed that Hoxa5, as an important transcription factor, bound to *Bax* promoter region and elevated the transcription activity of *Bax*. So, we suggested that Hoxa5 promoted white adipocyte apoptosis through mitochondrial pathway.

Akt and its downstream target mTOR form the core effector of the Akt/mTOR signaling pathway, which is involved in regulating cell proliferation, apoptosis, differentiation and intracellular energy metabolism [38]. It has been found in mammals that mTOR binds to other proteins to form two different complexes, mTORC1 and mTORC2. In contrast, mTORC1 is more sensitive to rapamycin and activates downstream S6 kinase (S6K) and eukaryotic translation initiation factor 4E binding protein 1 (4E-BP1) to regulate cell growth, apoptosis, protein synthesis and cell autophagy [39]. Recent studies have shown that the suppression of mTORC1 phosphorylation by specific inhibitor AZD8055 or rapamycin increases some apoptotic proteins of Bcl-2 family, and then amplifies apoptosis signal through the mitochondrial pathway, causing the downstream caspases cascade reaction to trigger apoptosis [39–41]. In support of these data, we detected the role of Akt/mTORC1/S6K1 signal pathway and used MK2206 and rapamycin, specific phosphorylation inhibitors of Akt and mTORC1. Results indicated that the promoting effect of Hoxa5 on adipocyte apoptosis was through inhibiting Akt/mTORC1/S6K1 signaling pathway.

**Figure 8:**
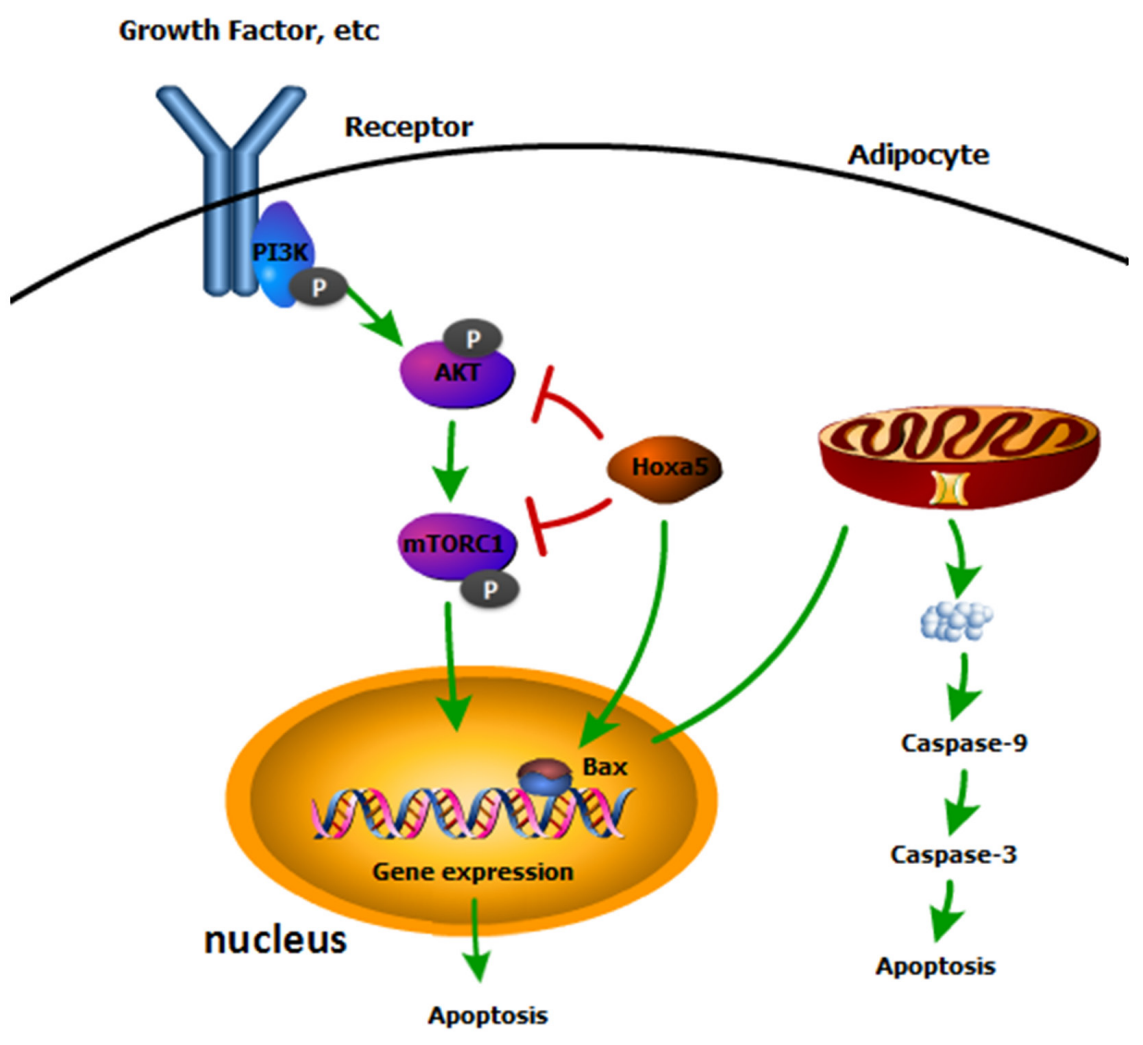
A summary of the regulation of Hoxa5 on mice white adipocyte apoptosis Our data indicated Hoxa5 increased white adipocyte apoptosis by inhibiting Akt/mTORC1 signaling pathway and Hoxa5 promoted transcription of *Bax* to promote mitochondrial apoptosis in white adipocytes of mice.

In summary, our study revealed that Hoxa5 promoted white adipocyte apoptosis by inhibiting Akt/mTORC1/S6K1 pathway. Also, we found that Hoxa5 elevated the transcription activity of *Bax*, which increased the mitochondrial apoptosis of adipocytes (Figure 8). Our findings might demonstrate a further understanding of regulatory mechanisms of adipocyte apoptosis and provide some novel approaches for therapies of obesity.

## MATERIALS AND METHODS

### Animal study

Six-week-old C57BL/6J male mice were purchased from the Laboratory Animal Center of the Fourth Military Medical University (Xi’an, China). Mice handling protocols were conducted following the guidelines and regulations approved by the Animal Ethics Committee of Northwest A&F University (Yangling, China). Mice were provided with *ad libitum* water and standard chow diet. The animal room was maintained under conditions of temperature at 25 ± 1 °C, humidity at 55 ± 5%, and a 12 h light and 12 h dark cycle. Seven-week-old mice were intraperitoneally injected either PBS or palmitate acid (PA, 1 μg/g) (Sigma, St. Louis, MO, USA) per 2 days for 2 weeks before dark cycle. Body weight and food intake were recorded per 2 days. iWAT and iBAT were removed on the 14^th^ day. After intraperitoneal injection of recombinant adenovirus overexpression vector of Hoxa5 (Ad-Hoxa5) and recombinant adenovirus interference vector of Hoxa5 (sh-Hoxa5) to mice for 6 days, iWAT were sampled for future studies.

### Primary adipocyte culture

Inguinal white adipose tissue was harvested, visible fibers and blood vessels were removed, and the adipose tissue was washed three times with a PBS buffer containing 200 U/mL penicillin (Sigma, St. Louis, MO) and 200 U/mL streptomycin (Sigma). White adipocytes were seeded onto 35 mm culture dishes at a density of 8×10^4^ cells/dish, and cells were maintained in medium containing Dulbecco's modified Eagle’s-H21 medium/Ham's F12 (DMEM/F12), 10% FBS (Gibco, Carlsbad, USA) and 1% penicillin-streptomycin and cells were incubated at 37 °C under a humidified atmosphere of 5% CO_2_ and 95% air until confluence. The medium was changed every other day. The adipocyte culture was carried out according to our previous publication [42].

### Chemical treatment and vectors infection

For the *in vitro* experiment, white adipocytes were treated with PA (250 nM) or bull serum albumin (BSA, 0.5%, control) for 24 h; serum-free medium or fetal bovine serum (FBS, 10%, control) medium for 12 h to induce apoptosis, and TM (1 μg/mL) for 12 h to create ERS. White adipocytes were infected with Ad-Hoxa5 or sh-Hoxa5 or 24 h or 48 h at the titer of 1×10^9^ IFU/mL. The recombinant adenovirus vector and interference vector of Hoxa5 (Ad-Hoxa5, sh-Hoxa5), adenovirus interference vector of Bax (sh-Bax) and control vectors were purchased from Gene Pharma (Shanghai, China). For signaling pathway study, the experimental procedure was as described in our previous reports in detail [43]. Briefly, white adipocytes were treated with Akt phosphorylation-specific inhibitor MK2206 (10 μM, Selleck, Houston, USA)and mTORC1 inhibitor rapamycin (10 μM, Selleck, Houston, USA) or DMSO (Promega, Madison, USA) for 6 h, and samples were assessed by Western blot.

### Apoptosis assessment

Cell viability was measured using Cell Counting Kit-8 (CCK-8, Vazyme, Nanjing, China) assay after incubation with PA or adenovirus vectors. After treatment with PA or adenovirus vectors, adipocytes were incubated for 30 min with Hoechst 33342 staining dye (Solarbio, Beijing, China), and washed three times with PBS. Cells were observed by using a Nikon TE2000-U microscope (Nikon, Tokyo, Japan).

The adipocyte apoptosis was measured by FITC-labeled annexin V/PI staining. An annexin V-FITC apoptosis assay kit (Vazyme, Nanjing, China) was used according to the manufacturer's protocol. Afterwards, cells were observed by using a Nikon TE2000-U microscope and the data was analyzed with Image J. The cell percentage of different apoptotic stages was determined by flow cytometry (BD FACScan; BD Biosciences) and data were analyzed using Cell Quest software (BD Biosciences).

The TUNEL assay was performed to detect the fragments of genome DNA by using *in situ* cell death detection kit (Roche, Switzerland). Adipocytes were infected with Ad-Hoxa5 or sh-Hoxa5 for 24 h. Then, adipocytes were trypsinized and suspended in 0.5 mL PBS, and added with 4% (v/v) paraformaldehyde in PBS to fix cells for 30 min at room temperature. Cells were permeabilized with 0.1% TritonX-100 on ice for 2 min and then stained by TUNEL fluorochrome. Cells were observed by using a Nikon TE2000-U microscope and the percentage of positive cells was analyzed with Image J.

### Mitochondrial membrane potential (ΔΨm) measurement

Fluorescent probe JC-1 (Beyotime, Nanjing, China) was used to estimate ΔΨm. The experimental procedure was as described previously [44]. Briefly, cells were incubated at 37 °C for 10 min with 5 μg/mL JC-1, then washed twice with PBS. Images were scanned by a fluorescence microscope (Nikon TE2000-U, Japan). The fluorescence intensity was analyzed with Image J. Also, adipocytes were trypsinized and suspended in 0.5 mL PBS for flow cytometer analysis. JC-1 was excited at 488 nm. JC-1 monomer signal (green) was analyzed at 525 nm and JC-1 aggregates signal (red) was analyzed at 590 nm. The ratio of red/green fluorescent intensity was calculated for the level of ΔΨm.

### Promoter reporter assay and dual luciferase reporter assay

The *Bax* promoter sequence was analyzed by using Genomatrix MatInspector. A fragment containing *Bax* - 5’ sequence -706 bp ~ +267 bp relative to the transcription initiation site was inserted into a pGL3-basic vector (Takara, Dalian, China), named pGL3-Bax. Mutant *Bax* reporter plasmids were generated using pGL3-Bax plasmid as a template, and a mutagenesis kit (Invitrogen, CA, USA) was used. HEK293T cells were co-transfected with Renilla plasmid, pGL3-basic or pGL3-Bax plasmid (control reporter), and *Hoxa5* overexpression plasmid (pc-Hoxa5). Cells were harvested 48 h after transfection and detected using the Dual-Luciferase Reporter assay system (Promega, Madison, WI, USA) [45].

### ChIP assay

White adipocytes were prepared for chromatin immunoprecipitation (ChIP) assay using a ChIP assay kit (Abcam, Cambridge, UK) according to the manufacturer's protocol. Primary antibodies of Hoxa5 (Abcam) or IgG (Abcam) were used. DNA–protein crosslinking complexes were collected, and purified DNA was subjected to qPCR with SYBR green fluorescent dye (Invitrogen, Carlsbad, CA, USA).

### Real-time quantitative PCR analysis

Total RNA was extracted from adipose tissues (iWAT and iBAT) or white adipocytes using TRIpure Reagent kit (Takara, Dalian, China) according to the manufacturer's instructions. 500 ng of total RNA was reverse transcribed using M-MLV reverse transcriptase kit (Takara, China). Primers were designed by Premier 5.0 and were synthesized by Invitrogen (Table 1, Shanghai, China). Real-time PCR amplification was performed in 25 μL reaction system containing specific primers and SYBR Premix (Vazyme, Nanjing, China) under specific amplification conditions. Amplification reactions were carried out in the ABI StepOne plus™ RT-PCR System (Carlsbad, CA). The levels of mRNA were normalized to Gapdh. 2^−ΔΔCt^ method was used to analyze the data.

**Table 1:** Primers for real-time quantitative PCR

Genes	Accession number	Primer sequences (5′ to 3′)
*Bax*	NM_007527.3	F: AGACAGGGGCCTTTTTGCTAC
		R: AATTCGCCGGAGACACTCG
*Bcl-2*	NM_177410.3	F: GCTACCGTCGTGACTTCGC
		R: CCAGCCTCCGTTATCCTGGA
*Bid*	NM_007544.3	F: GCCGAGCACATCACAGACC
		R: TGGCAATGTTGTGGATGATTTCT
*Bad*	NM_007522.3	F: TGAGCCGAGTGAGCAGGAA
		R: GCCTCCATGATGACTGTTGGT
*Caspase-3*	NM_009810.3	F: CTCGCTCTGGTACGGATGTG
		R: TCCCATAAATGACCCCTTCATCA
*Caspase-9*	NM_015733.5	F: GGCTGTTAAACCCCTAGACCA
		R: TGACGGGTCCAGCTTCACTA
*FasL*	NM_010177.4	F: CAGCCCATGAATTACCCATGT
		R: ATTTGTGTTGTGGTCCTTCTTCT
*Tnf-α*	NM_013693.3	F: CAGGCGGTGCCTATGTCTC
		R: CGATCACCCCGAAGTTCAGTAG
*Caspase-2*	NM_007610.2	F: TACTCCCACCGTTGAGCTGT
		R: CCGTAGCATCTGTGGATAGGC
*Caspase-8*	NM_001080126.1	F: TGCTTGGACTACATCCCACAC
		R: GTTGCAGTCTAGGAAGTTGACC
*Grp78*	NM_001163434.1	F: ACTTGGGGACCACCTATTCCT
		R: GTTGCCCTGATCGTTGGCTA
*Chop*	NM_007837.4	F: AAGCCTGGTATGAGGATCTGC
		R: TTCCTGGGGATGAGATATAGGTG
*Caspase-7*	NM_007611.2	F: AAGACGGAGTTGACGCCAAG
		R: CCGCAGAGGCATTTCTCTTC
*Caspase-12*	NM_009808.4	F: TAGGGGAAAGTGCGAGTTTCA
		R: GGGCCAATCCAGCATTTACCT
*Hoxa5*	NM_010453.5	F: CTCATTTTGCGGTCGCTATCC
		R: ATCCATGCCATTGTAGCCGTA
*Hoxa4*	NM_008265.3	F: CGGTGGTGTACCCCTGGAT
		R: GCTTAGGTTCGCCTCCGTTAT
*Hoxa7*	NM_010455.2	F: GCGCTTTTTAGCAAATATACGGC
		R: GGGATGTTTTGGTCGTAGGAG
*Hoxa9*	NM_010456.3	F: CCCCGACTTCAGTCCTTGC
		R: CCAGGAGCGCATATACCTGC
*GAPDH*	NM_008084.3	F: AGGTCGGTGTGAACGGATTTG
		R: GGGGTCGTTGATGGCAACA

### Western blot analysis

Protein from white adipocytes or white adipose tissue was extracted using lysing buffer. Protein concentration was determined using BCA Protein Assay kit (Beyotime, Nanjing, China). The experimental procedure was as described previously [46]. Briefly, Protein samples (30 μg) were separated by SDS-PAGE and transferred to PVDF nitrocellulose membranes (Millipore, USA), and blocked with 5% Skim Milk Powder/Tween 20/TBST at room temperature for 2 h. Then, primary antibodies against Bcl-2 (bs1511), Cleaved Caspase-9 (bs7070), Cleaved Caspase-3 (bs7004), Gapdh (ap0063), β-actin (ap0060) (Bioworld, CA, USA), Hoxa5 (ab140636), Bax (ab32503), Cytochrome C (ab53056), Akt (ab8805), p-Akt^Ser473^ (ab81283), mTOR (ab87540), p-mTORC1^Ser2448^(ab137133), and S6K1 (ab9366), p-S6K1^Thr389^ (ab2571) (Abcam, Cambridge, UK) were used to incubate the membranes at 4 °C overnight and the appropriate HRP-conjugated secondary antibodies (Boaoshen, China) were used for 2 h at room temperature. Akt phosphorylation-specific inhibitor MK2206 and mTORC1 inhibitor rapamycin were purchased from Selleck Chemical (USA). Proteins were visualized using chemiluminescent peroxidase substrate (Millipore, USA), and then the blots were quantified using ChemiDoc XRS system (Bio-Rad, USA).

### Statistics

Statistical analyses were performed using SAS v8.0 (SAS Institute, Cary, NC). Data were analyzed using one-way ANOVA procedure. Comparisons among individual means were made by Fisher's least significant difference (LSD). Data are presented as mean ± SEM. *P* < 0.05 was considered to be significant.
